# Molecular epidemiology of *Hepatitis delta virus* infection in Minas Gerais state from Brazil, an area outside the hyperendemic region of the Amazon Basin

**DOI:** 10.1590/0074-02760190074

**Published:** 2019-08-22

**Authors:** Cristiane FO Scarponi, Erna G Kroon, Deusilene S Vieira, Ana Paula Fernandes, Karina B Gomes, Bruno EF Mota

**Affiliations:** 1 Universidade Federal de Minas Gerais, Faculdade de Farmácia, Belo Horizonte, MG, Brasil; 2 Fundação Ezequiel Dias, Instituto Octávio Magalhães, Belo Horizonte, MG, Brasil; 3 Universidade Federal de Minas Gerais, Instituto de Ciências Biológicas, Belo Horizonte, MG, Brasil; 4 Fundação Oswaldo Cruz-Fiocruz, Porto Velho, RO, Brasil

**Keywords:** *Hepatitis D virus* genotype 1, seroprevalence, molecular epidemiology, Brazil

## Abstract

**BACKGROUND:**

*Hepatitis delta virus* (HDV) infections in hepatitis B virus (HBV) carriers are the most severe form of viral hepatitis. HDV prevalence is high in the Brazilian Amazon, but studies in other regions of the country are still scarce and often underestimated its prevalence by including a small numbers of individuals.

**OBJECTIVE:**

This study aimed to determine the serological prevalence of hepatitis D, the genotypes circulating and to evaluate the associated risk factors for acquisition of HDV in Minas Gerais state, Brazil.

**METHODS:**

We screened plasma samples (n = 498) from HBV chronic carriers for anti-HD antibodies using a commercial enzyme-linked immunosorbent assay (ELISA) kit. For those samples that were positive for anti-HD antibodies, we performed a reverse transcriptase (RT) nested-polymerase chain reaction (nested-PCR) in order to detect the viral genome and identify the viral genotypes circulating in the state.

**FINDINGS:**

The prevalence was 6.22% (31/498). Blood transfusion was the only risk factor associated with HDV infection [risk ratio: 3.73; 95% confidence interval (CI): 1.44 to 9.65]. For 26 anti-HD positive patients, HDAg gene sequences were determined and in all patients HDV genotype 1 was found.

**CONCLUSIONS:**

This study confirmed the circulation of HDV in Minas Gerais, an area previously considered non-endemic for hepatitis D in Brazil. The prevalence found in this study is much higher when compared to other studies performed in Brazil, probably because the population in our study was selected with minimal bias. Furthermore, in 26 anti-HD positive plasma samples, we were also able to detect the viral genome, indicating that these patients were experienced an active infection at the time of sample collection. These findings emphasise the importance of anti-HD testing in HBV infected individuals, which may contribute to this disease control in Brazil.

*Hepatitis D virus* (HDV or delta virus) is the single specie of the genus *Deltavirus*. HDV is a defective RNA virus that depends on the hepatitis B virus (HBV) infection to complete its life cycle. The helper function of HBV is to provide the surface antigen (HBsAg), which HDV use as its envelope protein. Delta virus is classified in at least eight genotypes (one to eight) based on the phylogenetic analysis of the partial sequence of the HDAg ORF or the complete genome (around 1.7 kbp). Globally, around 250 million individuals live with chronic HBV infection, of whom 15 million also have been superinfected with HDV.^1^

HDV infection is transmitted by the same routes as HBV infection, including sexual contact or parenteral transmission through use of intravenous drugs, surgical procedures or blood transfusion.^2^ Dual infection HDV/HBV may be acquired in two different ways. In one of these forms, called co-infection, HDV is acquired simultaneously with HBV, coursing as an acute infection, with minimal hepatic damage and total clearance of both viruses in the majority of cases (around 98%). In the super-infection condition, HDV infection is acquired by an individual already infected by HBV. Approximately 90% of these patients will develop chronic hepatitis with a high risk to experience severe clinical manifestations as fulminant hepatitis, liver decompensation, cirrhosis and hepatocellular carcinoma. Unlike several effective therapeutic options available for HBV infection, HDV-HBV dual infection can only be treated with high doses of interferon alpha.^3^

Chronic HBV infection is marked by persistent positivity of HBsAg and can be broadly divided into three different profiles: (i) active hepatitis, which correlates with HBeAg positivity, elevation of plasmatic transaminases and a decrease in HBV DNA levels, (ii) the inactive carrier state, which is characterised by conversion of HBeAg to anti-HBe and low or undetectable levels of plasmatic transaminases and HBV-DNA and (iii) the immunotolerant phase, which is characterised by high levels of HBV DNA and low levels of plasmatic transaminases. HDV infection is diagnosed by the detection of total or IgM anti-HD antibodies and/or viral RNA in plasma, which is a marker of active viral replication.^2^

In Brazil, HDV infection is well documented in the Amazon Basin, where its prevalence is one of the highest in the world.^4^ ^,^ ^5^ On the other hand, there is scant and possibly outdated information on prevalence of HDV infections in other parts of Brazil.^6^ ^,^ ^7^ ^,^ ^8^ A recent study disclosed the results of a national survey in Brazil, based on the prevalence of anti-HD antibodies. Prevalence values of 1.7% were reported in the Southeastern region of Brazil and 1.96% among individuals (1/51) of Minas Gerais state.^9^ However, this study did not access the disease status (active viral replication or past infection), virus genotyping and included a limited number of individuals.

Besides epidemiological issues and their impact in control policies, genotyping of HDV is important due to different clinical profiles associated with each genotype. HDV genotype 1 is the most commonly found in Brazil, and was associated with a wide range of clinical manifestations. HDV genotype 3, which is restricted to the Amazon Basin, is associated with a fulminant form of hepatitis, known as Labrea fever.^10^ One study described the circulation of HDV genotype 8 in Maranhão state, northeastern of Brazil.^11^ However, so far, there is no study describing the molecular epidemiology of HDV in other Brazilian regions, mainly in the dense populated states of the Southeastern region, including Minas Gerais. Therefore, this study aimed to investigate the prevalence, associated factors and the molecular epidemiology of HDV infection in Minas Gerais state, Brazil.

## MATERIALS AND METHODS

*Ethical statements* - All procedures of this study were performed in accordance with standard ethical rules and were approved by the Research Ethics Committee of Universidade Federal de Minas Gerais (protocol number CAAE, 14253013.7.0000.5149).

*Study design and subjects* - This cross-sectional epidemiological study enrolled patients attending the Ezequiel Dias Foundation (FUNED), from May 2012 to August 2013. This foundation is the Central Public Health Reference Laboratory, responsible for performing quantification of HBV viral load, after serological diagnosis of this infection in Minas Gerais state. So, all patients that were diagnosed with HBV in the state have their samples sent to FUNED, regardless of the presence of clinical symptoms.

Minas Gerais state is located in the Southeastern region of Brazil. It is the fourth largest state in the country, which occupies a land area of 586,521,235 km^2^, being divided into 853 municipalities. The estimated population is about 19,600,000 habitants, being the second most populous state in the country (accessed at www.censo2010.ibge.gov.br).

Only samples from chronic HBV carriers (positive HBsAg for more than six months) were included in the study (n = 498). Demographic, laboratorial and clinical data were obtained from questionnaires answered by the physician responsible for each studied patient.

*Serological analyses* - Plasma samples, obtained from blood collected in EDTA tube, were stored at -20ºC until serological testing. Samples were tested in two independent experiments for the detection of total anti-HD antibodies, using a commercial enzyme immunoassay kit (ETI-AB-2-DELTAK, DiaSorin, Saluggia, Italy), according to the manufacturer’s instructions.

*Molecular analyses* - For samples with detectable total anti-HD antibodies, RNA was extracted using the QIAamp Viral RNA Mini Kit (Qiagen, Hilden, Germany), according to the manufacturer’s instructions. The extracted RNA was previously denatured at 95ºC for 5 min then it was reverse transcribed and the cDNA obtained was amplified in a one-step reverse transcription polymerase chain reaction (RT-PCR), using the QuantiTect Probe RT-PCR kit (Qiagen, Germany), using the outer primers forward 853 IU 5’ CGGATGCCCAGGTCGGACC 3’ and reverse 1302 OD 5’ GGATTCACCGACAAGGAGAG 3’.^1^ The product of the first reaction was used in the second reaction (Nested-PCR) employing inner primers HDV-E 5’ GAGATGCCATGCCGACCCGAAGAG 3’ and HDV-A 5’ GAAGGAAGGCCCTCGAGAACAAGA 3’.^2^ Reactions conditions were: 95ºC for 5 min followed by 30 cycles of 95ºC for 30 s, 55ºC for 30 s and 72ºC for 1 min with a final step of 72ºC for 10 min. The PCR products were analysed by electrophoresis in 1% agarose gels and visualised under UV light, using SYBR Safe stain (Thermo Fisher Scientific, Waltham, MA, United States). The amplified DNA was purified using Wizard® SV Gel and PCR Clean-Up System (Promega Corporation, USA), prior to sequencing in ABI Prism 3730 Genetic DNA Analyser using the ABI Prism BigDye Terminator Cycle Sequencing Ready Reaction kit (Applied Biosystems, USA).

*Phylogenetic reconstruction* - The sequences generated were aligned with standard sequences stored in Gen Bank database (https://www.ncbi.nlm.nih.gov/genbank/) using the algorithm MUSCLE implemented in the EMBL web page (http://www.ebi.ac.uk/Tools/msa/muscle/). The phylogeny was reconstructed using the maximum likelihood method in PHYML algorithm.^3^ The model of nucleotide substitution used was selected by prior analysis using smart model selection (SMS), implemented in PHYML.^4^ The resulting trees were visualised using FigTree v.1.2.2 software (http://tree.bio.ed.ac.uk/software/figtree/).

*Statistical analysis* - Sample size estimation was based on 5% prevalence rate of HDV, with 2% margin of error and 95% confidence level, by StatCalc software, v.7.1.4.0 (Epi Info, CDC, Georgia, USA). Data were analysed using STATA software, v.12.0 (Stata Corp LP, Texas, USA). Continuous variables were described by median and interquartile range (IIQ), being analysed by Mann-Whitney test. Categorical variables were represented as frequency distribution and percentage, being compared using the Chi-square test or Fisher’s exact test. Those variables that presented p-value below 0.2 (in univariate analysis) were included into the multivariate logistic regression model (Reverse stepwise). Significant differences were considered for p-value < 0.05.

## RESULTS

*HDV prevalence in Minas Gerais state, Brazil* - Of the 498 samples tested, 31 were positive for total anti-HD antibodies, resulting in an overall prevalence of HDV infection of 6.22% among the chronic HBV carriers. The difference of median age of patients with positive and negative HDV antibody showed a significant association [37 years old (IIQ: 28-44) and 42 (IIQ: 33-52), respectively], in a preliminary univariate analysis (p = 0.033), but no evidence of association was observed after stratification by age range. The proportion of men and women were similar when compared positive and negative HDV antibody patients (p = 0.872). The proportion of HDV-infected men (54.8%) and women (56.2%) were similar among the chronic HBV carriers. Most HBV patients, as well as HDV-positive antibody patients had a low educational level (≤ 8 years), measured by years of schooling. However, presence of anti-HD antibodies could not be associated with age range, gender or with the educational level of the patients included in the study (Table I).

**TABLE I:** Sociodemographic characteristics of chronic hepatitis B virus (HBV) patients infected with *Hepatitis D virus* (HDV) in the state of Minas Gerais, Brazil

Variable	Total anti-HD antibody		p-value
	Nº negative (%)	Nº positive (%)	
Age group (years)			0.068^*a,c*^
0-19	10 (2.14)	0 (0.00)	
20-39	182 (38.97)	20 (64.52)	
40-59	217 (46.47)	9 (29.03)	
≥ 60	58 (12.42)	2 (6.45)	
Gender			0.872^*b*^
Male	263 (56.32)	17 (54.84)	
Female	204 (43.68)	14 (45.16)	
Educational level^*d*^			0.647^*a*^
0 years	3 (1.65)	0 (0.00)	
≤ 8 years	105 (57.69)	9 (69.23)	
> 8 years	74 (40.66)	4 (30.77)	

*a*: two-tailed *p*-value from Fisher’s exact test; *b*: two-tailed p-value from Pearson’s chi-square test; *c*: entered into logistic regression for multivariate analysis; *d*: total patients differ due to missing data.

*Clinical profile of HDV positive cases* - Regarding the clinical profile of chronic HBV carriers, there is no difference between the two groups (HBV mono-infected *versus* HDV/HBV dual infection). More than half of positive HDV/HBV patients (57.14%) included in our study were inactive carriers, compared to 67.33% of inactive carriers in negative HDV population, while active hepatitis was reported for 19.02% of negative HDV patients *versus* 21.43% of HDV infected individuals (p = 0.198). The frequency of positive anti-HBe marker (25/27, 92.59%) was higher than positive HBeAg (2/25, 7.41%) in the infected HDV patients. However, this pattern was not significantly different from the frequencies among uninfected HDV patients (p = 0.402). Alanine aminotransferase (ALT) levels in HDV infected individuals were within normal reference values (5-60 U/L) for 19 patients (70.37%), but up to two times higher than the reference value in five patients (18.52%) and more than two times higher than normal limit (ULN) in three patients (0.11%); a similar pattern was detected in uninfected HDV patients (p = 0.098). These data are shown in Table II.

**TABLE II:** Comparison of clinical and laboratory profiles between serological *Hepatitis D virus* (HDV) positive and negative in hepatitis B virus (HBV) chronic carriers in Minas Gerais state, Brazil

Variable	Total anti-HD antibody		p-value
	Nº negative (%)	Nº positive (%)	
IgM anti-HBc^*d*^			0.705^*a*^
Negative	277 (88.50)	17 (94.44)	
Positive	36 (11.50)	1 (5.55)	
Anti-HBe^*d*^			0.402^*a*^
Negative	59 (14.43)	2 (7.40)	
Positive	350 (85.57 )	25 (92.59)	
HBeAg^*d*^			0.755^*a*^
Negative	385 (88.51)	25 (92.59)	
Positive	50 (11.49)	2 (7.40)	
HCV/HIV coinfection			1.000^*a*^
Negative	396 (93.84)	30 (96.77)	
Positive	26 (6.16)	1 (3.23)	
Chronic HBV stages^*d*^			0.198^*a,c*^
Inactive carrier	301 (67.33)	16 (57.14)	
Active hepatitis B	85 (19.02)	6 (21.43)	
Cirrhosis	32 (7.16)	5 (17.86)	
Imunnotolerant	29 (6.49)	1 (3.57)	
ALT levels^*d*^			0.098^*a,c*^
≤ UNL	351 (77.65)	19 (70.37)	
> UNL, ≤ 2x UNL	88 (19.47)	5 (18.52)	
> 2x UNL	13 (2.88)	3 (11.11)	
Cirrhotic			0.078^*b,c*^
No	394 (92.71)	26 (83.87)	
Yes	31 (7.29)	5 (16.13)	
Realised biopsy^*d*^			0.501^*a*^
No	385 (90.16)	27 (96.43)	
Yes	42 (9.84)	1 (3.57)	

*a*: two-tailed p-value from Fisher’s exact test; *b*: two-tailed p-value from Pearson’s chi-square test; *c*: entered into logistic regression for multivariate analysis; *d*: total patients differ due to missing data; ALT: alanine aminotransferase; UNL: upper normal limit alanine aminotransferase (ALT > 60 U/L).

HBV-DNA was undetectable in 12.90% of samples from patients with dual HDV-HBV infection. In 74.07% of these samples, HBV-DNA load was in the range of 20 to 2,000 IU/mL, and in 13.03% viral load was higher than 2,000 IU/mL, with median viral titer of 2.04 log IU/mL (IIQ: 1.30-3.25). The median HBV-DNA titer among negative HDV samples was 2.10 log IU/mL (IIQ: 1.30-3.08) and was not significantly different between the two groups (p = 0.906, data not shown).

Regarding antiviral therapy, 24 out of the 31 infected HDV patients (77.42%) reported to be treatment-naïve for HBV infection at the time of the study. Seven patients had a prior history of antiviral treatment, four with entecavir (4/7, 57.14%), one with lamivudine, one with tenofovir and one with adefovir (14.28% each one). None of them was previously subjected to therapy with Interferon.

*Risk factors for HDV infection in Minas Gerais state, Brazil* - The analysis of potential risk factors for HDV acquisition in Minas Gerais showed that there is no significant association between HDV infection and the risk factors traditionally described for these infections, such as contact with HBV infected individuals, use of injectable medicines or intravenous drug use, multiple sexual partners and medical procedures like surgery, organ transplantation and hemodialysis. Additionally, no association was found between the detection of anti-HD and any type of institutionalisation or occupation of the patients. However, blood transfusion or blood products recipients was the single variable displaying statistical association with HDV infection among the studied sample (p = 0.004, Table III).

To exclude possible confounding factors, all variables that displayed a p-value < 0.2 in the univariate analysis were included into the logistic regression model. After multivariate analysis, only the variable blood or blood products transfusion history remained statistically associated with HDV infection (p = 0.007). The risk ratio of HDV infection for individuals who have received blood products is 3.73 compared to those patients that have not received blood transfusion (95% CI: 1.44 to 9.65).

**TABLE III:** Analysis of risk factors for *Hepatitis D virus* (HDV) infection in hepatitis B virus (HBV) chronic carriers, Minas Gerais state, Brazil

Variable^*d*^	Total anti-HD antibody		p-value
	Nº negative (%)	Nº positive (%)	
Dwelling contact with HBV carrier			
No	122 (69.71)	9 (81.82)	0.317^*a*^
Yes	53 (30.29)	2 (18.18)	
Sexual contact with HBV carrier			
No	112 (84.85)	6 (85.71)	0.680^*a*^
Yes	22 (15.15)	1 (14.29)	
Occupational contact with HBV carrier			
No	160 (91.95)	9 (90.00)	0.582^*a*^
Yes	14 (8.05)	1 (10.00)	
Institutionalised patient			
No	263 (56.32)	17 (54.84)	0.872^*b*^
Yes	204 (46.68)	14 (45.16)	
Injectable medicines			
No	198 (61.49)	13 (56.52)	0.637^*b*^
Yes	124 (38.51)	10 (43.48)	
Tattoo/piercing			
No	284 (87.93)	19 (82.61)	0.508^*a*^
Yes	39 (12.07)	4 (17.39)	
Accident with biological material			
No	310 (93.66)	20 (86.96)	0.197^*a,c*^
Yes	21 (6.34)	3 (13.04)	
Inhaled drugs users			
No	292 (90.40)	20 (86.96)	0.484^*a*^
Yes	31 (9.60)	3 (13.04)	
Acupuncture history			
No	295 (94.55)	21 (87.50)	0.163^*a,c*^
Yes	17 (5.45)	3 (12.50)	
Blood transfusion			
No	299 (90.06)	17 (70.83)	0.004*a* ^*,b,c,e*^
Yes	33 (9.94)	7 (29.17)	
Injectable illicit drugs			
No	302 (93.21)	20 (86.96)	0.224^*a*^
Yes	22 (6.79)	3 (13.04)	
Surgery procedure			
No	206 (64.78)	11 (50.00)	0.163^*b,c*^
Yes	112 (35.22)	11 (50.00)	
Dental procedure			
No	112 (39.58)	10 (45.46)	0.588^*b*^
Yes	171 (60.42)	12 (54.54)	
Multiple sex partners			
No	173 (57.67)	11 (57.89)	0.984^*b*^
Yes	127 (42.33)	8 (42.10)	
Dialysis procedure			
No	313 (93.71)	21 (87.50)	0.211^*a*^
Yes	21 (6.29)	3 (12.50)	
Organ transplant			
No	323 (95.56)	19 (86.36)	0.089^*a,c*^
Yes	15 (4.44)	3 (13.64)	

*a*: two-tailed p-value from Fisher’s exact test; *b*: two-tailed p-value from Pearson’s chi-square test; *c*: entered into logistic regression for multivariate analysis; *d*: total patients differ due to missing data; *e*: statistically significant (p < 0.05).

*HDV nucleic acid detection and phylogenetic analysis* - Among the 31 plasma samples that showed anti-HD antibodies, 27 (87.10%) were also positive for HDV RNA by reverse transcriptase (RT) nested-PCR assay, displaying a specific amplicon of 404 bp, corresponding to a partial fragment of HDAg gene (from nucleotide 883 to 1,287 in HDV genome M84917.1). Sequencing and phylogenetic analysis of 26 of these samples showed that all the HDV strains circulating in the Minas Gerais state belong to the genotype 1, as supported by high values of bootstrap analysis (Figure). More specifically, our sequences clustered together with viruses detected both from Brazil (Patient_HBV_HDV_HIV_Brazil) as well from the US (US1 and US2). For the missing sample it was not possible to determine the genotype. The sequences used in this study were deposited in GenBank under accession numbers MK101320 through MK101345.

**Figure:**
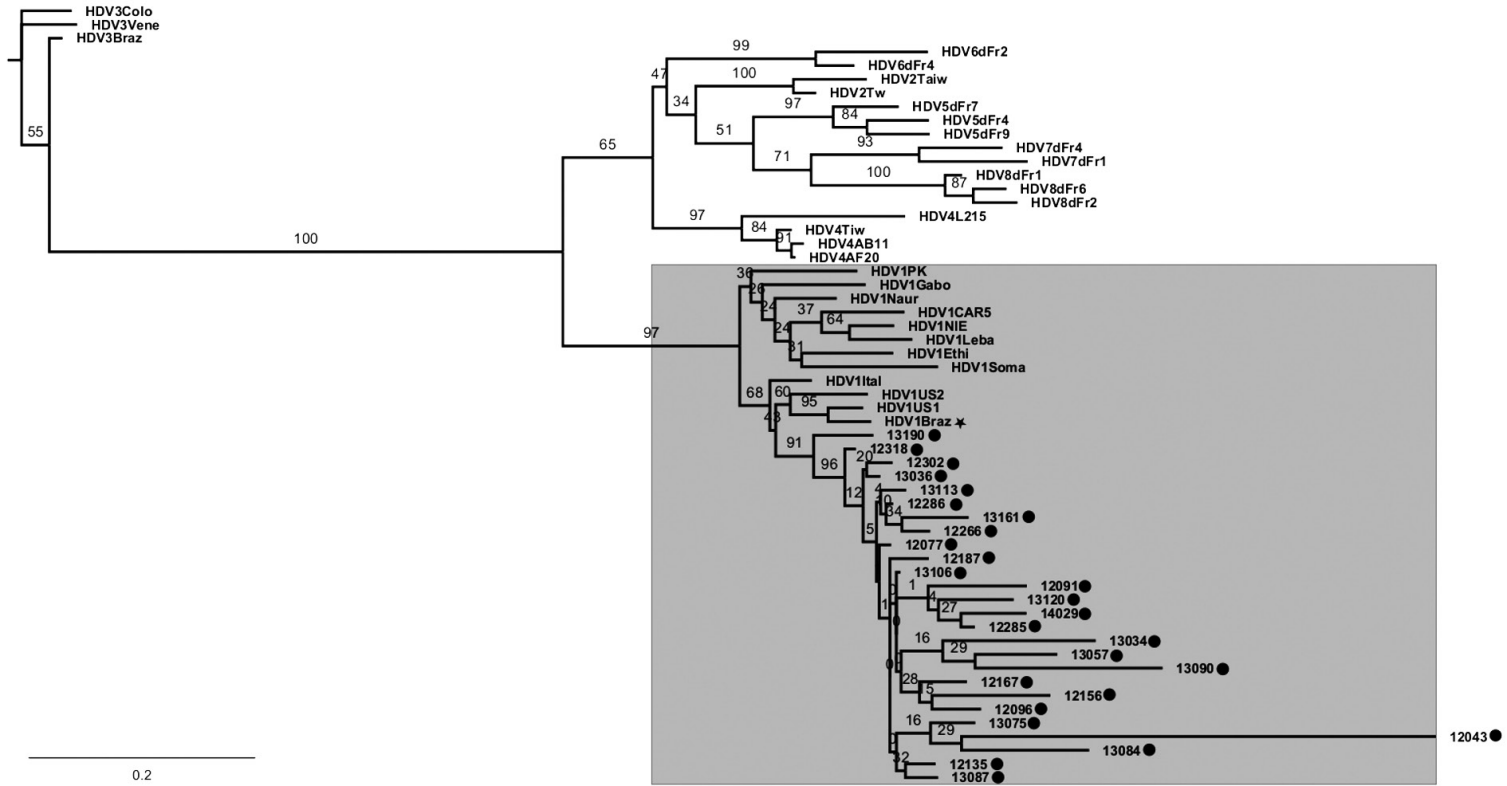
Phylogenetic analysis of *Hepatitis D virus* (HDV) strains circulating in Minas Gerais state, Brazil. Phylogenetic reconstruction was based in the partial sequence of the HDAg gene. The sequences generated (marked with a circle) were aligned with standard sequences deposited in GenBank database, using the algorithm MUSCLE. The model of nucleotide substitution used (GTR + gamma) was selected using PHYML smart model selection (SMS). The phylogeny was reconstructed using the maximum likelihood method in PHYML algorithm. The resulting trees were visualised using FigTree v.1.2.2 software. The GenBank accession number for the standard sequences used are: HDV1PK (JN400348.1), HDV1Gabo (EU035520.1), HDV1Naur (M58629), HDV1CAR5 (JX888135.1), HDV1NIE (JX888121.1), HDV1Ethi (U81989.1), HDV1Soma (U81988.1), HDV1US1 (D01075), HDV1US2 (L22066), HDV1Braz (HQ686061), HDV1Ital (X04451), HDV1Leba (M84917), HDV2Tw (AF104264), HDV2Taiw (U19598), HDV3Vene (AB037948.1), HDV3Colo (EU287872.1), HDV3Braz (KC590319.1), HDV4AB11 (AB118847.1), HDV4AF20 (AF209859.1), HDV4L215 (AB088679), HDV4Tiw (AF018077), HDV5dFr7 (AX741154), HDV5dFr4 (AX741149), HDV5dFr9 (AX741159), HDV6dFr4 (AX741164), HDV6dFr2 (AJ583887), HDV7dFr4 (AX741164), HDV7dFr1 (AJ583885), HDV8dFr1 (AM183330.1), HDV8dFr2 (AM183327.1), HDV8dFr6 (AX741169). Highlighted in gray is the clade corresponding to HDV genotype 1. The other Brazilian HDV-1 sequence deposited in Gen Bank (HDV1Braz) is marked with a star.

## DISCUSSION

This is the first study, to our knowledge, to describe the occurrence of active delta virus infection in Minas Gerais, a state outside the Amazon Basin, in Brazil. Moreover, we have outlined a more detailed epidemiological scenario of HDV infection in a southeastern state of Brazil, with a target population selected with the minimum bias. Our findings confirm the existence of HDV infection in the state, with 6.22% overall frequency (31/498) for total anti-HD antibodies in the studied population.

The results shown herein differ from most studies performed in this region of Brazil, where no HDV cases were detected.^5^ ^,^ ^6^ ^,^ ^7^ In a recent national survey, the general prevalence of anti-HD antibodies in chronic HBV carriers in Brazil was 3.2% and in Minas Gerais state this prevalence was 1.96%, far lower than the prevalence found in our study.^9^ The limited sample size and the different criteria for inclusion of participants in other studies could explain, at least in part, the discrepancies found in the HDV prevalence among these studies, leading to underestimated prevalences in general.

Most notably, our results are in agreement with estimations from other non-endemic countries. In a recent study, conducted in Northern California (USA), 42 of the 499 chronic HBV carriers (8%) tested positive for anti-HD antibodies.^12^. Other data reported in the literature indicated a prevalence of HDV of 8.3% in Egyptian patients,^13^ 5.5% in Belgium,^14^ 6.5% in Guangdong, China,^15^ 7.1% in South London^16^ and 7.7% in Tehran, Iran.^17^

Another relevant finding is the clinical status of the HDV infected individuals in our study. Traditionally, dual infection of HDV/HBV is marked by high levels of the plasmatic enzyme alanine aminotransferase (ALT) and decreased HBV viral loads.^18^ However, the majority of the HDV infected individuals in this study presented ALT levels under normal limits (70.37%, Table II) and HBV viral loads comparable to that of HBV mono-infected individuals. However, in order to more accurately estimate the long-term effects of HDV-HBV infection in Minas Gerais state, a follow-up study would be more appropriate.

Aiming to identify the probable risk factors for acquisition of HDV infection, the associations between classic behavioral variables for transmission of viral hepatitis versus HDV status were analysed in the studied population.^18^ ^,^ ^19^ ^,^ ^20^ Our results pointed that blood transfusion history was the single risk factor independently associated with HDV infection among chronic HBV patients in Minas Gerais. This finding is consistent with other studies, in which blood transfusion proved to be a risk factor for HDV infection.^21^ Our results showed an association almost four times higher between transfused patients versus not transfused, for hepatitis D infection (Table III). Currently, screening with HBV markers provides a high level of safety in the prevention of HDV infections in blood banks. However, contaminated blood may be eventually transfused, because of false negative detection of HBV infections, due to the ability of HDV to suppress HBV replication, or to the immunological window, during blood bank screening.

An important consideration regarding the treatment of hepatitis D is the availability of a single alternative ― interferon alpha (IFN-α), which has antiviral activity by inhibiting mRNA and protein synthesis, besides promoting widespread activation of the immune system.^3^ In accordance with prior studies, nucleoside and nucleotide analogs used in the treatment of HBV infection are ineffective against HDV. Given that active HDV infection was not previously suspected, based in the underreported or outdated epidemiological data, it is not surprising that none of the patients have been previously tested for HDV and consequently none of them have received IFN-α. Based on our survey, among those receiving treatment with other antiviral drugs, three patients have not reached a sustained virological response or changed medication during treatment (data not shown). Thus, in Minas Gerais, HDV infected patients are not receiving the most appropriate therapy for HBV-HDV infections.

Viral RNA was detected in 27 out of 31 (87.10%) patients with positive total anti-HD antibodies. This finding indicates that most of the patients are experiencing an active infection with delta virus. The amplified cDNA was successfully sequenced for 25 patients. Phylogenetic analyses using the maximum likelihood method showed that all these patients are infected with HDV genotype 1 (Figure). Specifically, the sequences generated in this work are in the same clade that strains isolated in Brazil from a patient with a triple HBV-HDV-HIV infection^22^ and strains derived from North American patients with fulminant liver disease.^23^ ^,^ ^24^ These three HDV strains, in its turn, are closely related to European HDV.^22^ ^,^ ^23^ ^,^ ^24^ This fact suggests that Brazilian genotype 1 strains (and other New World genotype 1 strains) may have derived from European HDV-1 strains. This genotype is spread worldwide and may lead to a wide variety of clinical presentations in infected patients. In our study, most patients presented clinically as inactive carriers, with anti-HBeAg seroconversion and low levels of plasmatic ALT and HBV genomic DNA. This is an important aspect, since the suspicion of HDV infection is often based on the presence of advanced liver disease in individuals that came from endemic areas (such as the Brazilian Amazon).

*In conclusion* - The findings described herein demonstrate for the first time that HDV genotype 1 is circulating in Minas Gerais state, Brazil and that blood transfusion is the most important risk factor for acquisition of HDV infection in the state. This study contributes to improve the current understanding of the epidemiological and clinical aspects of hepatitis D in Brazil, emphasising the relevance of future research in other areas beyond the Amazon Basin. Moreover, it suggests for the need of better management of diagnosis, treatment and follow-up of chronic HBV carriers and implementation of focused public health actions and control measures in the localities with high number of cases.
